# Procalcitonin, Presepsin, Endocan, and Interleukin-6 in the Early Diagnosis of Neonatal Sepsis—A Prospective Study

**DOI:** 10.3390/diagnostics15111341

**Published:** 2025-05-26

**Authors:** Maura-Adelina Hincu, Liliana Gheorghe, Cristina Dimitriu, Luminita Paduraru, Gabriela Zonda, Dan-Constantin Andronic, Ingrid-Andrada Vasilache, Luiza-Maria Baean, Dragos Nemescu

**Affiliations:** 1 Department of Mother and Child Care, University of Medicine and Pharmacy “Grigore T. Popa”, 700115 Iasi, Romania; 2 Surgical Department, Faculty of Medicine, University of Medicine and Pharmacy “Grigore T. Popa”, 700115 Iasi, Romania; 3 Department of Morpho-Functional Sciences II, University of Medicine and Pharmacy “Grigore T. Popa”, 700115 Iasi, Romania; 4 Department of Neonatology, Centre Hospitalier Public du Cotentin, 50100 Cherbourg-en-Cotentin, France; ildikozonda@yahoo.com; 5 Department of Fundamental Disciplines, Faculty of Midwifes and General Asistans, University of Medicine and Pharmacy “Carol Davila”, 020021 Bucharest, Romania

**Keywords:** early-onset sepsis, procalcitonin, presepsin, interleukin-6, endocan, diagnostic accuracy, cut-off values

## Abstract

**Background/Objectives**: Neonatal early-onset sepsis (EOS) is a life-threatening condition, and numerous efforts have been invested in identifying the most promising biomarkers for its detection. In this prospective cohort study, we aimed to determine the diagnostic accuracy and optimal cut-off values of procalcitonin (PCT), presepsin, endocan, and interleukin (IL)-6 determined from the neonatal serum (0–12, 24–48, and 72–96 h), and umbilical blood cord for the diagnosis of EOS. **Methods**: A total of 122 patients were included in this study and were divided into two groups: group 1 (sepsis, n = 68 patients) and group 2 (without sepsis, n = 54 patients). Maternal and neonatal characteristics were assessed using descriptive statistics. Logistic regressions were used to evaluate the association between various biomarkers and the presence of EOS and to adjust for potential confounders. Using sensitivity analysis and Youden’s index from the ROC curve, the biomarkers’ diagnostic accuracy and optimal cut-off values were obtained. **Results**: PCT at 0–12 and 24–48 h of life exhibited the best diagnostic performance, with sensitivities (Ses) of 75% and 76.5% and specificities (Sps) above 74%. Presepsin demonstrated excellent performance at 24–48 h, with Ses of 68.42%, and Sps of 88.89%. IL-6 and endocan achieved modest results for the detection of EOS. **Conclusions**: PCT and presepsin measured at early neonatal timepoints demonstrated high diagnostic accuracy and favorable sensitivity–specificity balance for predicting EOS.

## 1. Introduction

Neonatal early-onset sepsis (EOS) is characterized by the presence of a pathogenic bacterial species in a blood or cerebrospinal fluid culture collected within the first 72 h of life and continues to impact the neonatal morbidity and mortality rates worldwide [1]. However, various regions of the world have different mortality rates attributed to neonatal infections depending on specific factors related to demographic profile of the population, medical infrastructure, and financial resources [2]. Across the European continent, recent reports have highlighted persistent disparities in infant mortality rates, with significantly higher figures in Eastern Europe compared to Western Europe. For example, in 2023, infant mortality was reported at 3.3 deaths per 1000 live births in Western Europe, while rates in countries such as Romania and Slovakia reached 5.7 per 1000 live births [3]. These differences may reflect variations in access to timely and accurate diagnosis and management of neonatal sepsis.

A positive blood culture serves as the definitive diagnostic method, although the confirmation of its results occurs within a 36–48 h period. Despite the presence of particular signs and symptoms, fewer than 1% of newborns suspected of having sepsis yield a positive blood culture [4]. Although real-time polymerase chain reaction (PCR) assays could establish the diagnosis of neonatal sepsis faster than blood cultures, these are not routinely available [5]. The presence of sepsis biomarkers capable of notifying clinicians for the early detection of neonatal sepsis has the potential to improve both the immediate and future outcomes for actual sepsis patients, while simultaneously minimizing the unnecessary and detrimental use of preventative antibiotics [6].

Both C-reactive protein (CRP) and procalcitonin (PCT) are traditionally used serum markers for the diagnosis of neonatal sepsis. Other studied biomarkers include interleukin 6 (IL-6), presepsin, and endocan, all of which have exhibited significant variability in terms of diagnostic accuracy of the disease. For example, a recent systematic review and meta-analysis that evaluated the diagnostic accuracy of biomarkers determined maternal serum, umbilical cord blood, and neonatal serum for EOS and reported a pooled sensitivity (Se) of 79%, and a pooled specificity (Sp) of 91% for PCT, as well as a pooled Se of 83%, and a pooled Sp of 87% for IL-6, both determined from the umbilical cord blood [7]. Moreover, the authors reported a pooled Se of 82%, and an Sp of 86%, for presepsin determined from the neonatal serum. However, the diagnostic accuracy of combined biomarkers for the diagnosis of EOS is poorly studied, especially in various demographic regions where racial and ethnic disparities could influence their performance [8].

The primary aim of this prospective cohort study was to determine the diagnostic accuracy of PCT, presepsin, endocan, and IL-6 determined from the neonatal serum (0–12 h, 24–48 h, and 72–96 h) and umbilical blood cord for the diagnosis of EOS. The secondary aim of this study was to assess the diagnostic accuracy of the individual biomarkers for EOS using optimal cut-off values.

## 2. Materials and Methods

This prospective cohort study included patients diagnosed with or without a diagnosis of EOS who were born at “Cuza voda” Clinical Hospital of Obstetrics and Gynecology, Iasi, Romania, between 2019 and 2024. Inclusion criteria comprised the following: singleton neonates admitted to the neonatal intensive care unit (NICU) with or without suspected EOS diagnosis that was later confirmed according to the criteria proposed by the European Medicines Agency (EMA) [9], with a gestational age of 28 weeks or higher, for whom the serum samples could be collected at 0–12 h, 24–48 h, and 72–96 h post-birth, with available umbilical cord blood and/or maternal serum samples collected at birth and for whom informed consent was obtained from participants. For all participants in this study, the samples were processed if the EOS diagnosis was confirmed by positive hemocultures.

The exclusion criteria comprised severe congenital malformations or genetic syndromes, major perinatal complications unrelated to infection (i.e., hypoxic-ischemic encephalopathy-HIE, severe intraventricular hemorrhage-IVH), inadequate or missing serum sample volumes for biomarker analysis, mothers with known immunological, autoimmune diseases or chronic infections, and lack of informed consent.

Ethical approval for conducting this study was obtained from the Institutional Ethics Committees of the “Grigore T. Popa” University of Medicine and Pharmacy, Iasi (No. 175/17.04.2022) and “Cuza voda” Clinical Hospital of Obstetrics and Gynecology (No. 5750/09.05.2022 and No. 1405/02.02.2023).

The following data were recorded from the medical records: maternal and perinatal data (inadequate prenatal care, antepartum hemorrhage, antepartum antibiotics, corticosteroid therapy, maternal white blood cell count, CRP, fibrinogen, endocan levels, comorbidities such as gestational diabetes, pre-eclampsia, thrombophilia, etc.), delivery and perinatal factors (meconial amniotic fluid, duration of ruptured membranes, clinical or histological chorioamnionitis, amniotic fluid culture, vaginal secretion cultures, urine culture, lochia culture), birth and neonatal characteristics (sex, type of delivery, gestational age at birth, birth weight, intrauterine growth restriction, need for neonatal resuscitation, Apgar scores at 1 and 5 min, respiratory distress syndrome, and blood cultures), neonatal manifestations (fever, tachycardia, hypotension, inotropic support, renal impairment, metabolic acidosis, thrombocytopenia, hypoglycemia or hyperglycemia, persistent pulmonary hypertension), and neonatal complications (pneumothorax, pulmonary hemorrhage, feeding intolerance, intraventricular hemorrhage, retinopathy of prematurity).

Apart from these data, we determined a series of biochemical markers: white blood cell-WBC count (0–12 h, 24–48 h, and 72–96 h), fibrinogen (0–12 h, 24–48 h, and 72–96 h), CRP levels from cord blood, serum CRP values (0–12 h, 24–48 h, and 72–96 h), PCT levels from cord blood, serum PCT values (0–12 h, 24–48 h, and 72–96 h), IL-6 levels from cord blood and from serum of the neonates (0–12 h, 24–48 h, and 72–96 h), presepsin levels from the cord blood and from serum of the neonates (0–12 h, 24–48 h, and 72–96 h), as well as endocan levels from the cord blood and from the neonatal serum (0–12 h, 24–48 h, and 72–96 h).

The blood samples from mothers and newborns were stored in duplicates at −20 °C until processing. The PCT levels were determined using sandwich ELISA (Human PCT ELISA Kit, Elabscience, Houston, TX, USA) and reported as pg/mL. IL-6 determination from serum was performed according to the manufacturer’s indications using sandwich ELISA (Human IL-6 ELISA Kit, Elabscience, USA) and the serum levels were expressed as pg/mL. The same method was used to determine the serum levels of endocan (MyBioSource Human Endocan ELISA kit, San Diego, CA, USA), which were expressed as pg/mL, as well as presepsin serum levels (MyBioSource Human Presepsin ELISA Kit, San Diego, CA, USA).

We performed a sample size calculation that would be able to detect a 20% difference in the mean values of the evaluated biomarkers between the septic and non-septic groups, considering a two-tailed alpha value of 0.05 and a power of 80%. The estimated sample size was 17 patients per group, totaling 34 participants. A total of 122 patients were included in this study and for statistical purposes were divided into two groups: group 1 (sepsis, n = 68 patients) and group 2 (without sepsis, n = 54 patients).

Descriptive statistics were used to assess both maternal and neonatal characteristics. Specifically, the Student’s *t*-test was used to compare continuous variables with a normal distribution between groups, while the Wilcoxon rank-sum test was used to compare non-normally distributed continuous variables. On the other hand, categorical variables were compared between groups using chi-square test.

In the second stage of the analysis, we conducted multivariable logistic regression in order to evaluate the association between various biomarkers and the presence of EOS. We first performed univariable logistic regression analyses to estimate the crude association between each biomarker and EOS. Subsequently, multivariable logistic regression models were constructed to adjust for potential confounders, including gestational age at birth, duration of membrane rupture, and previous maternal antibiotic exposure. These covariates were selected based on their known or potential influence on neonatal sepsis risk and biomarker expression.

Adjusted odds ratios (aORs) and 95% confidence intervals (CIs) were reported to quantify the effect size of each biomarker on the odds of EOS. Confounding was assessed by comparing the crude and adjusted estimates; a change of ≥10% in the biomarker coefficient was considered indicative of confounding.

To evaluate the diagnostic accuracy of individual biomarkers in diagnosing EOS, we calculated standard performance metrics, including sensitivity (Se), specificity (Sp), positive predictive value (PPV), negative predictive value (NPV), accuracy, and the area under the receiver operating characteristic curve (AUC).

For each biomarker, optimal cut-off values were determined using Youden’s index derived from the ROC curve. All analyses were conducted using Stata version 18.5 (StataCorp, College Station, TX, USA), and a two-tailed *p*-value < 0.05 was considered statistically significant.

## 3. Results

Mothers whose newborns developed sepsis presented with significantly higher rates of pre-eclampsia (8.82% versus 0%, *p* = 0.025) and thrombophilia (11.76% versus 0%, *p* = 0.009) than mothers whose newborns did not develop sepsis (Table 1). Also, these patients had significantly higher rates of positive vaginal cultures (20.59% versus 7.41%, *p* = 0.016). On the other hand, mothers whose newborns did not develop sepsis presented significantly higher rates of prolonged rupture of membranes (74.07% versus 38.24%, *p* < 0.001) in comparison with their counterparts. Also, this category of patients benefited significantly more frequently from antepartum antibiotic therapy (62.96% versus 35.29%, *p* = 0.002) and corticosteroid therapy (37.04% versus 14.71%, *p* = 0.004).

Table 2 comprises a comparison of maternal biomarkers between the two groups, and our results indicated that only mean CRP values (15.94 ± 18.49 versus 8.22 ± 10.18 mg/L, *p* = 0.007) at admission and endocan levels (1279.15 ± 407.06 versus 970.01 ± 726.69 pg/mL, *p* = 0.007) were significantly higher for mothers whose newborns developed sepsis in comparison with the CRP values in the control group.

Neonates with a septic state experienced significantly higher rates of fever (8.82% versus 0%, *p* = 0.025), tachycardia (11.76% versus 0%, *p* = 0.009), hypotension (23.53% versus 3.70%, *p* = 0.002), and needed significantly more inotropic support (23.53% versus 3.70%, *p* = 0.020) in comparison with neonates without this condition (Table 3). Duration of parenteral feeding (8.59 ± 5.89 versus 6.00 ± 5.70 days, *p* = 0.0159) and antibiotic treatment (7.44 ± 3.79 versus 4.81 ± 2.56, *p* < 0.001) were significantly higher for septic neonates in comparison with controls.

Also, as expected, this category of patients presented significantly higher rates of metabolic acidosis (26.47% versus 3.70%, *p* = 0.001), pneumothorax (23.53% versus 3.70%, *p* = 0.002), pulmonary hemorrhage (14.71% versus 3.70%, *p* = 0.043), and feeding intolerance (20.59% versus 7.41%, *p* = 0.041). No significant differences regarding death rates were encountered between the two groups (*p* = 0.40).

The paraclinical characteristics of the evaluated cohort indicated that neonates with sepsis presented significantly lower values of hemoglobin (*p* = 0.003), hematocrit (*p* = 0.003), and WBC in the first 12 h of life (*p* = 0.0001) compared with the values encountered in neonates without sepsis (Table 4).

Also, this category of patients presented with significantly higher levels of fibrinogen at 24–48 h (*p* < 0.001), and at 72–96 h (*p* < 0.001), I/T ratios at 24–48 h (*p* = 0.0001) and 72–96 h (*p* < 0.001), and CRP serum levels determined from umbilical cord (*p* = 0.02), 0–12 h (*p* < 0.001), 24–48 h (*p* < 0.001), and 72–96 h (*p* < 0.001).

A comparison between the levels of biomarkers is presented in Table 5. Our data indicated that presepsin serum levels determined at 72–96 h of life (*p* = 0.08) and endocan levels determined from umbilical cord blood (*p* = 0.255) did not significantly differ between the evaluated groups. On the other hand, values of PCT, IL-6, presepsin, and endocan were significantly higher for neonates with sepsis.

In Table 6, we present the results from the univariate and multivariable logistic regression to evaluate the effect of predictors and covariates on the EOS occurrence. The results from univariate logistic regressions indicated that serum values of PCT at 0–12 h (*p* < 0.001), 24–48 h (*p* < 0.001), and 72–96 h (*p* = 0.017), IL-6 determined from umbilical cord (*p* = 0.032) and serum at 0–12 h (*p* = 0.001), presepsin determined from umbilical cord (*p* = 0.003) and from serum at 0–12 h (*p* < 0.001) and 24–48 h (*p* < 0.001), as well as serum endocan levels at 0–12 h (*p* = 0.007) and at 24–48 h (*p* = 0.011) were significant predictors for EOS.

For PCT, across all time points, adjusted ORs remained close to unadjusted ORs, changing by less than approximately 7%, which indicates a minimal confounding effect of covariates. Across all the remaining biomarkers, adjustment for gestational age, ROM duration, and antibiotic therapy duration produced only an overall minimal confounding effect (between 0.05 and 6.5%).

In Table 7, we present the results from the testing for significant individual biomarkers for the prediction of EOS. PCT at 0–12 h (Se—75%, Sp—85.19%, and accuracy—79.51%) and 24–48 h (Se—76.47%, Sp—74.07%, and accuracy—75.41%) achieved the best performance metrics in terms of sensitivity, specificity, and accuracy for EOS detection.

Even though IL-6 determined from the umbilical cord and from the neonatal serum at 0–12 h achieved better overall accuracy than PCT, it was characterized by low sensitivities (22.22% and 44.44%), and high specificity (100% and 96.3%).

Presepsin determined from the neonatal serum at 24–48 h achieved a moderate performance when used to diagnose EOS, with an Se of 68.42%, Sp of 88.89%, and accuracy of 80.43%. Also, endocan serum levels achieved the poorest performance for the detection of EOS (Se: 22.22–24%, Sp: 85.19–88.89%, and accuracy: 65.82–66.67%).

Finally, in Table 8, we present the calculated cut-offs for individual biomarkers that offer the best balance between sensitivity and specificity for EOS diagnosis. Our results indicated that PCT at 0–12 h (cutoff 7.81: Se—75%, Sp—85%, AUC value—0.80, J—0.60) and 24–48 h (cutoff 15.585: Se—74%, Sp—85%, AUC value—0.79, J—0.59) and presepsin at 24–48 h (cutoff 31.698: Se—68%, Sp—93%, AUC value—0.81, J—0.61) obtained the best values of Youden index.

## 4. Discussion

This prospective cohort study aimed to evaluate the diagnostic accuracy of PCT, presepsin, endocan, and IL-6 assessed at designated neonatal and maternal timepoints for the early detection of EOS in neonates. We also assessed the best cut-off values for achieving the highest diagnostic performance of individual biomarkers at specific timepoints. Our findings emphasize the clinical significance of PCT and presepsin as primary diagnostic tools of EOS while highlighting limitations in the predictive performance of endocan and IL-6, especially when used individually.

PCT is a prohormone of calcitonin, lacking hormonal activity, encoded by the CALC-I gene on chromosome 11, and secreted during sepsis and inflammation [10]. Its secretion commences within 2 h post-stimulation, reaches its peak at 12–24 h, and exhibits a half-life of around 24 h [11]. Our results indicated that among all the biomarkers assessed, PCT—particularly at 0–12 and 24–48 h of life—exhibited the best diagnostic performance, with sensitivities of 75% and 76.5%, specificities above 74%, and AUC values approaching 0.80. The estimated optimal cut-offs using the Liu approach validated the significant discriminative power of PCT, with Youden indices of 0.60 and 0.59 for the initial two timepoints, hence strengthening its utility in clinical triage and decision-making.

These findings align with the prior evidence suggesting that PCT rises rapidly in systemic infections and may serve as sensitive marker for EOS. A recent literature review performed by Eschborn et al., which evaluated the diagnosis performance of PCT and CRP for neonatal sepsis, indicated a mean sensitivity of 73.6% and a mean specificity of 82.8% of PCT for EOS sepsis, higher than the diagnostic performance of CRP (mean sensitivity: 65.6% and mean specificity: 82.7%) [12]. Moreover, a recent prospective cohort study by Rautela et al. that evaluated the diagnostic accuracy for EOS of IL-27 in comparison with CRP and PCT serum levels, indicated that PCT showed the highest sensitivity (82.93%) for EOS diagnosis, followed by IL-27 (sensitivity of 78.05%) and CRP (sensitivity of 73.17%) [13].

Presepsin, a marker of monocyte activation and innate immune response [14], demonstrated excellent performance at 24–48 h, with a Youden index of 0.61 and an AUC of 0.81. These values are comparable to PCT and suggest a strong role for presepsin in complementing clinical assessment, particularly in cases with ambiguous clinical presentation. Although its umbilical cord and 0–12 h values showed reduced sensitivity, its specificity remained high, offering potential utility in ruling-out sepsis as demonstrated by other studies [15,16,17].

IL-6 determined from umbilical cord blood or neonates’ serum at 0–12 h of life, while biologically plausible as a sepsis marker as reported in several studies [18,19], showed limited sensitivity (22–44%), despite high specificity (93–100%). This reflects its brief half-life and highlights the critical importance of timing in biomarker sampling. These findings caution against relying on IL-6 alone as a screening tool, especially in settings where sepsis evolves sub-clinically.

Last, but not least, endocan demonstrated low sensitivity, moderate specificity, and modest overall diagnostic accuracies at 0–12 and 24–48 h after birth (65.82% and 66.67%). Its diagnostic performance (Youden indices ≤ 0.48) suggests limited clinical utility when used individually, despite its statistical associations in univariate analyses with EOS. Another prospective study conducted in Romania on a cohort of 59 patients indicated that for a calculated optimal threshold value of 1.62 ng/mL, serum endocan presented a sensitivity of 88% and a specificity of 50% for the diagnosis of EOS [20]. On the other hand, another study did not indicate endocan as a promising diagnostic marker for late-onset neonatal sepsis [21].

The literature data indicate tumor necrosis factor alpha (TNF-α), progranulin or neopterin as potential alternative diagnostic biomarkers. The cut-off value ranges of TNF-α for the diagnosis of EOS between 1.7 and 70 pg/mL have a sensitivity of 66–78% and a specificity of 41–76% according to recent reports [22,23,24]. A threshold of 18.94 pg/mL demonstrated a sensitivity of 79% and a specificity of 81% [23]. Given its moderate accuracy, this biomarker is considered a more reliable indicator for late-onset sepsis [23].

Another study pointed out that a cut-off value >37.89 ng/mL of progranulin achieved a diagnostic accuracy of 0.786 for EOS, with a sensitivity of 94.3%, specificity of 51.5%, positive predictive value of 61.7%, and negative predictive value of 91.7% [25]. Moreover, the combination of PRGN with PCT increased the diagnostic accuracy for EOS to 0.987 [25].

Last but not least, Shokry and colleagues evaluated the utility of neopterin in diagnosing EOS in full-term neonates. At a cut-off value of 499 nmol/L, this biomarker achieved an AUC value of 0.91 along with a sensitivity of 91%, specificity of 84.7%, positive predictive value of 91.9%, and negative predictive value of 88% [26]. A separate study conducted on both pre-term and full-term neonates reported excellent diagnostic accuracy for EOS, with an AUC of 0.992 at a threshold value of 100.3 nmol/L [27].

One strength of this study lies in the rigorous evaluation of confounding variables, including gestational age, duration of membrane rupture, and duration of maternal antibiotic therapy. Adjusting for these variables yielded minimal changes in effect estimates across all biomarkers (generally < 7%), indicating that these biomarkers retained independent predictive value. The stability of adjusted odds ratios further supports the robustness of the diagnostic signal, particularly for PCT and presepsin. Notably, these covariates may function more as effect modifiers, given their biological plausibility in influencing neonatal immune response and sepsis risk. Other strengths of this study include its prospective design, and a certain diagnosis of EOS based on blood culture confirmation.

On the other hand, the limitations of this study include small sample size, limited variability, inclusion of neonates admitted to the NICU, and lack of stratification based on specific gestational-age groups.

Further studies could use machine learning-based methods to explore the potential of individual biomarkers for EOS diagnosis along with specific maternal and neonatal risk factors. This could highlight the predictive performance of individual markers in specific clinical situations and at various time-points. Moreover, further validation of these algorithms would allow clinicians to establish the best approach for diagnosis EOS as soon as the first days of neonatal life, thus reducing the burden of unnecessary antibiotic administration.

## 5. Conclusions

PCT and presepsin measured at early neonatal timepoints—particularly at 0–12 h and 24–48 h—demonstrated high diagnostic accuracy and a favorable sensitivity–specificity balance for predicting EOS.

IL-6 and endocan may serve as adjunctive markers, though their performance is dependent on precise timing and clinical context.

Further multicenter validation and investigation of combined biomarker models are warranted to establish standardized, high-performance diagnostic algorithms for EOS.

## Figures and Tables

**Table 1:** Maternal clinical characteristics.

Variable	Sepsis (Group 1, 68 Patients)	No Sepsis (Group 2, 54 Patients)	*p*-Value
Inadequate prenatal care (%)	29.41	14.81	0.057
Antepartum hemorrhage (%)	2.94	3.70	0.814
Antepartum antibiotics (%)	35.29	62.96	0.002
Corticosteroid therapy (%)	14.71	37.04	0.004
Gestational diabetes (%)	5.88	3.70	0.580
Preeclampsia (%)	8.82	0.00	0.025
Thrombophilia (%)	11.76	0.00	0.009
Rupture of membranes > 18 h (%)	38.24	74.07	<0.001
Amniotic fluid culture (positive) (%)	44.12	25.93	0.235
Vaginal secretions culture (positive) (%)	20.59	7.41	0.016

**Table 2:** Comparison of maternal biomarkers.

Variable	Sepsis (n = 68) Mean ± SD	No Sepsis (n = 54) Mean ± SD	Mean Difference (0–1)	*p*-Value
WBC (×10^9^/L)	12.13 ± 4.65	12.35 ± 4.78	0.22	0.797
CRP (mg/L)	15.94 ± 18.49	8.22 ± 10.18	−7.72	0.007
Fibrinogen (mg/dL)	392.03 ± 147.41	380.81 ± 45.54	−11.21	0.591
Endocan (pg/mL)	1279.15 ± 407.06	970.01 ± 726.69	−309.14	0.04

Table legend: WBC—white blood cells; CRP—C-reactive protein; SD—standard deviation;

**Table 3:** Neonatal clinical characteristics and complications.

Variable	Sepsis (n = 68 Patients)	No Sepsis (n = 54 Patients)	*p*-Value
Sex (female) (%)	47.06	33.33	0.126
Type of delivery (cesarean) (%)	64.71	62.96	0.842
Gestational age at birth (weeks)	32.53 ± 4.60	32.04 ± 4.01	0.5358
Birthweight (g)	2001.47 ± 974.61	1907.04 ± 786.93	0.5645
Apgar score at 1 min	5.91 ± 2.37	5.81 ± 2.11	0.8144
Apgar score at 5 min	7.06 ± 1.66	6.81 ± 1.69	0.4252
SGA (%)	29.41	22.22	0.06
Fever (%)	8.82	0.00	0.025
Tachycardia (%)	11.76	0.00	0.009
Hypotension (%)	23.53	3.70	0.002
Inotropic support ≥ 1 day (%)	23.53	3.70	0.020
Renal impairment (%)	3.70	0.00	0.110
Metabolic acidosis (%)	26.47	3.70	0.001
Thrombocytopenia (%)	23.53	3.70	0.002
Hypoglycemia (%)	38.24	37.04	0.892
Hyperglycemia (%)	11.76	7.41	0.422
Persistent pulm. hypertension (%)	8.82	0.00	0.025
Blood culture positive (%)	17.65	0.00	0.032
Positive tracheal aspirate	61.76	0.00	0.001
Pneumothorax (%)	20.59	3.70	0.006
Pulmonary hemorrhage (%)	14.71	3.70	0.043
Feeding intolerance (%)	20.59	7.41	0.041
Intraventricular hemorrhage (%)	11.76	7.41	0.422
Retinopathy of prematurity (%)	11.76	11.11	0.124
Days of hospitalization	30.32 ± 26.59	36.93 ± 27.67	0.1834
Days of parenteral feeding	8.59 ± 5.89	6.00 ± 5.70	0.0159
Days of antibiotic therapy	7.44 ± 3.79	4.81 ± 2.56	<0.001
Death (%)	7.35	3.70	0.40

Legend: SGA—small for gestational age.

**Table 4:** Neonatal paraclinical characteristics.

Variable	Sepsis (n = 68) Mean ± SD	No Sepsis (n = 54) Mean ± SD	*p*-Value
Hemoglobin (g/dL)	15.01 ± 2.72	16.47 ± 2.56	0.0031
Hematocrit (%)	46.00 ± 8.31	50.36 ± 7.76	0.0037
Platelets (×10^3^/µL)	211.47 ± 92.11	260.74 ± 81.04	0.0025
WBC 0–12 h (×10^3^/µL)	12.64 ± 7.27	18.69 ± 9.64	0.0001
WBC 24–48 h (×10^3^/µL)	14.18 ± 8.10	16.91 ± 9.93	0.0964
WBC 72–96 h (×10^3^/µL)	13.71 ± 8.66	13.23 ± 7.08	0.7400
Fibrinogen 24–48 h (mg/dL)	311.62 ± 128.20	198.48 ± 65.88	<0.0001
Fibrinogen 72–96 h (mg/dL)	325.56 ± 129.07	211.37 ± 66.06	<0.0001
I/T ratio 0–12 h	12.68 ± 71.43	0.20 ± 0.14	0.2022
I/T ratio 24–48 h	0.29 ± 0.16	0.19 ± 0.13	0.0001
I/T ratio 72–96 h	0.20 ± 0.10	0.12 ± 0.06	<0.0001
CRP from umbilical cord (mg/L)	10.04 ± 5.67	7.15 ± 4.40	0.0279
CRP 0–12 h (mg/L)	33.39 ± 36.03	9.91 ± 9.25	<0.0001
CRP 24–48 h (mg/L)	35.24 ± 24.28	7.79 ± 6.08	<0.0001
CRP 72–96 h (mg/L)	21.74 ± 27.64	6.56 ± 4.13	<0.0001

Legend: WBC—white blood cells; I/T—immature to totals ratio; CRP—C-reactive protein.

**Table 5:** Comparisons of biomarkers’ values between the evaluated groups.

Variable (Unit)	Sepsis (Mean ± SD)	No Sepsis (Mean ± SD)	*p*-Value
PCT (ng/mL)—umbilical cord	3.95 ± 10.24	0.47 ± 1.15	0.0151
PCT (ng/mL)—0–12 h	21.65 ± 23.77	3.63 ± 6.86	<0.001
PCT (ng/mL)—24–48 h	27.07 ± 23.93	7.69 ± 9.09	<0.001
PCT (ng/mL)—72–96 h	5.18 ± 8.45	1.72 ± 1.82	0.0037
IL-6 (pg/mL)—umbilical cord	529.01 ± 875.04	120.87 ± 135.08	0.0013
IL-6 (pg/mL)—0–12 h	939.83 ± 1570.05	138.81 ± 161.44	<0.001
IL-6 (pg/mL)—24–48 h	878.11 ± 2418.42	178.46 ± 281.67	0.0381
IL-6 (pg/mL)—72–96 h	907.60 ± 2455.13	118.59 ± 162.62	0.0200
Presepsin (ng/mL)—umbilical cord	12.39 ± 7.37	7.05 ± 5.90	0.0012
Presepsin (ng/mL)—0–12 h	24.65 ± 17.56	9.44 ± 7.36	<0.001
Presepsin (ng/mL)—24–48 h	41.50 ± 20.79	14.27 ± 13.46	<0.001
Presepsin (ng/mL)—72–96 h	19.52 ± 13.24	13.99 ± 11.19	0.087
Endocan (pg/mL)—umbilical cord	2406.79 ± 836.05	2714.40 ± 1137.98	0.255
Endocan (pg/mL)—0–12 h	3454.43 ± 1406.68	2606.93 ± 1149.44	0.0058
Endocan (pg/mL)—24–48 h	3619.92 ± 1055.10	2865.90 ± 1301.20	0.0108
Endocan (pg/mL)—72–96 h	3415.90 ± 914.09	2796.41 ± 1378.45	0.048

Legend: PCT—procalcitonin; IL-6—interleukin-6; SD—standard deviation.

**Table 6:** Results from univariate and multivariable logistic regression to evaluate the effect of predictors and covariates on the EOS occurrence.

Variable	Covariate	Odds Ratio	Std. Error	*p*-Value	95% CI
PCT—umbilical cord	-	1.1497	0.1193	0.179	0.938–1.409
PCT—umbilical cord	Gestational age	1.109	0.0897	0.201	0.946–1.300
PCT—umbilical cord	ROM duration	1.1348	0.0975	0.141	0.959–1.343
PCT—umbilical cord	AB therapy duration	1.1514	0.1501	0.279	0.892–1.487
PCT—0–12 h	-	1.1373	0.0287	<0.001	1.082–1.195
PCT—0–12 h	Gestational age	1.1394	0.0291	<0.001	1.084–1.198
PCT—0–12 h	ROM duration	1.1447	0.0306	<0.001	1.086–1.206
PCT—0–12 h	AB therapy duration	1.13	0.0291	<0.001	1.074–1.188
PCT—24–48 h	-	1.112	0.0244	<0.001	1.065–1.161
PCT—24–48 h	Gestational age	1.1148	0.025	<0.001	1.067–1.165
PCT—24–48 h	ROM duration	1.1342	0.0285	<0.001	1.080–1.191
PCT—24–48 h	AB therapy duration	1.1355	0.0293	<0.001	1.079–1.195
PCT—72–96 h	-	1.171	0.0775	0.017	1.029–1.333
PCT—72–96 h	Gestational age	1.2178	0.0947	0.011	1.046–1.418
PCT—72–96 h	ROM duration	1.1911	0.0868	0.016	1.033–1.374
PCT—72–96 h	AB therapy duration	1.2218	0.0905	0.007	1.057–1.413
IL-6—umbilical cord	-	1.003	0.0014	0.032	1.000–1.006
IL-6—umbilical cord	Gestational age	1.0025	0.0013	0.06	0.999–1.005
IL-6—umbilical cord	ROM duration	1.0033	0.0015	0.023	1.000–1.006
IL-6—umbilical cord	AB therapy duration	1.0036	0.0017	0.031	1.000–1.007
IL-6—0–12 h	-	1.0044	0.0013	0.001	1.002–1.007
IL-6—0–12 h	Gestational age	1.0047	0.0015	0.001	1.002–1.008
IL-6—0–12 h	ROM duration	1.0047	0.0014	0.001	1.002–1.007
IL-6—0–12 h	AB therapy duration	1.0043	0.0021	0.044	1.000–1.008
IL-6—24–48 h	-	1.0004	0.0003	0.178	0.999–1.001
IL-6—24–48 h	Gestational age	1.0005	0.0004	0.158	0.999–1.001
IL-6—24–48 h	ROM duration	1.0021	0.0015	0.153	0.999–1.005
IL-6—24–48 h	AB therapy duration	1.0005	0.0003	0.156	0.999–1.001
IL-6—72–96 h	-	1.0005	0.0004	0.183	0.999–1.001
IL-6—72–96 h	Gestational age	1.0007	0.0004	0.133	0.999–1.002
IL-6—72–96 h	ROM duration	1.0032	0.0016	0.048	1.000–1.006
IL-6—72–96 h	AB therapy duration	1.0006	0.0004	0.162	0.999–1.001
Presepsin—umbilical cord	-	1.1202	0.0426	0.003	1.040–1.207
Presepsin—umbilical cord	Gestational age	1.1358	0.047	0.002	1.047–1.232
Presepsin—umbilical cord	ROM duration	1.1399	0.0463	0.001	1.053–1.234
Presepsin—umbilical cord	AB therapy duration	1.137	0.0477	0.002	1.047–1.234
Presepsin—0–12 h	-	1.0967	0.0265	<0.001	1.046–1.150
Presepsin—0–12 h	Gestational age	1.1018	0.0266	<0.001	1.051–1.155
Presepsin—0–12 h	ROM duration	1.1037	0.0275	<0.001	1.051–1.159
Presepsin—0–12 h	AB therapy duration	1.103	0.0269	<0.001	1.051–1.157
Presepsin—24–48 h	-	1.0784	0.0161	<0.001	1.047–1.111
Presepsin—24–48 h	Gestational age	1.0898	0.0191	<0.001	1.053–1.128
Presepsin—24–48 h	ROM duration	1.086	0.0175	<0.001	1.052–1.121
Presepsin—24–48 h	AB therapy duration	1.0844	0.0177	<0.001	1.050–1.120
Presepsin—72–96 h	-	1.0383	0.0232	0.092	0.994–1.085
Presepsin—72–96 h	Gestational age	1.0316	0.024	0.181	0.986–1.080
Presepsin—72–96 h	ROM duration	1.0474	0.0249	0.052	1.000–1.097
Presepsin—72–96 h	AB therapy duration	1.0102	0.024	0.67	0.964–1.058
Endocan—umbilical cord	-	0.9997	0.0003	0.232	0.9991–1.0002
Endocan—umbilical cord	Gestational age	0.9997	0.0003	0.199	0.9991–1.0002
Endocan—umbilical cord	ROM duration	0.9997	0.0003	0.196	0.9991–1.0003
Endocan—umbilical cord	AB therapy duration	0.9996	0.0003	0.093	0.9991–1.0002
Endocan—0–12 h	-	1.0005	0.0002	0.007	1.0001–1.0009
Endocan—0–12 h	Gestational age	1.0005	0.0002	0.017	1.0001–1.0009
Endocan—0–12 h	ROM duration	1.0005	0.0002	0.011	1.0001–1.0009
Endocan—0–12 h	AB therapy duration	1.0005	0.0002	0.017	1.0001–1.0009
Endocan—24–48 h	-	1.0005	0.0002	0.011	1.0001–1.0009
Endocan—24–48 h	Gestational age	1.0005	0.0002	0.018	1.0001–1.0009
Endocan—24–48 h	ROM duration	1.0005	0.0002	0.032	1.0001–1.0008
Endocan—24–48 h	AB therapy duration	1.0005	0.0002	0.04	1.0001–1.0009
Endocan—72–96 h	-	1.0004	0.0002	0.052	1.0000–1.0008
Endocan—72–96 h	Gestational age	1.0004	0.0002	0.099	1.0000–1.0008
Endocan—72–96 h	ROM duration	1.0004	0.0002	0.101	1.0000–1.0008
Endocan—72–96 h	AB therapy duration	1.0005	0.0002	0.029	1.0000–1.0009

Legend: PCT—procalcitonin; IL-6—interleukin-6; ROM—rupture of membranes; AB—antibiotic therapy; CI—confidence intervals.

**Table 7:** Performance of significant individual biomarkers for EOS diagnosis.

Biomarker	Timepoint	Sensitivity	Specificity	PPV	NPV	Accuracy
PCT	0–12 h	75.00%	85.19%	86.44%	73.02%	79.51%
PCT	24–48 h	76.47%	74.07%	78.79%	71.43%	75.41%
PCT	72–96 h	52.94%	55.56%	60.00%	48.39%	54.10%
IL-6	Cord	22.22%	100.00%	100.00%	79.41%	80.56%
IL-6	0–12 h	44.44%	96.30%	80.00%	83.87%	83.33%
Presepsin	Cord	30.43%	85.19%	46.67%	74.19%	68.83%
Presepsin	0–12 h	44.00%	92.59%	73.33%	78.12%	77.22%
Presepsin	24–48 h	68.42%	88.89%	81.25%	80.00%	80.43%
Endocan	0–12 h	24.00%	85.19%	42.86%	70.77%	65.82%
Endocan	24–48 h	22.22%	88.89%	50.00%	69.57%	66.67%

Legend: PCT—procalcitonin; IL-6—interleukin-6; PPV—positive predictive value; NPV—negative predictive value.

**Table 8:** Performance of significant individual biomarkers for EOS occurrence.

Biomarker	Timepoint	Cutoff	Se	Sp	AUC	Youden Index (J)
PCT (ng/mL)	0–12 h	7.81	0.75	0.85	0.80	0.60
PCT (ng/mL)	24–48 h	15.585	0.74	0.85	0.79	0.59
PCT (ng/mL)	72–96 h	3.505	0.38	0.89	0.64	0.27
IL-6 (pg/mL)	Cord	309.928	0.44	0.93	0.69	0.37
IL-6 (pg/mL)	0–12 h	119.684	0.89	0.70	0.80	0.59
Presepsin (ng/mL)	Cord	8.789	0.65	0.77	0.76	0.42
Presepsin (ng/mL)	0–12 h	7.0825	1.00	0.56	0.78	0.56
Presepsin (ng/mL)	24–48 h	31.698	0.68	0.93	0.81	0.61
Endocan (pg/mL)	0–12 h	2795.009	0.76	0.67	0.71	0.43
Endocan (pg/mL)	24–48 h	2789.249	0.85	0.63	0.74	0.48

Legend: PCT—procalcitonin; IL-6—interleukin-6; Se—sensitivity; Sp—specificity; AUC—area under the curve.

## Data Availability

Dataset available on request from the authors.

## Abbreviations

The following abbreviations are used in this manuscript:

Abbreviation	Full Term
AB therapy	Antibiotic Therapy
aOR	Adjusted Odds Ratio
AUC	Area Under the Curve
CI	Confidence Interval
CRP	C-Reactive Protein
ELISA	Enzyme-Linked Immunosorbent Assay
EMA	European Medicines Agency
EOS	Early-Onset Sepsis
HIE	Hypoxic-Ischemic Encephalopathy
IL-6	Interleukin-6
IL-27	Interleukin-27
I/T ratio	Immature-to-Total Neutrophil Ratio
IVH	Intraventricular Hemorrhage
NICU	Neonatal Intensive Care Unit
NPV	Negative Predictive Value
OR	Odds Ratio
PCR	Polymerase Chain Reaction
PCT	Procalcitonin
PPV	Positive Predictive Value
ROM	Rupture of Membranes
SD	Standard Deviation
Se	Sensitivity
SGA	Small for Gestational Age
Sp	Specificity
WBC	White Blood Cells
